# eEF1A2 siRNA Suppresses MPP^+^-Induced Activation of Akt and mTOR and Potentiates Caspase-3 Activation in a Parkinson's Disease Model

**DOI:** 10.1155/2023/1335201

**Published:** 2023-04-03

**Authors:** Kawinthra Khwanraj, Athinan Prommahom, Permphan Dharmasaroja

**Affiliations:** ^1^Department of Anatomy, Faculty of Science, Mahidol University, Bangkok, Thailand; ^2^Chakri Naruebodindra Medical Institute, Faculty of Medicine Ramathibodi Hospital, Mahidol University, Samut Prakan, Thailand

## Abstract

The tissue-specific protein eEF1A2 has been linked to the development of neurological disorders. The role of eEF1A2 in the pathogenesis of Parkinson's disease (PD) has yet to be investigated. The aim of this study was to determine the potential neuroprotective effects of eEF1A2 in an MPP^+^ model of PD. Differentiated SH-SY5Y cells were transfected with eEF1A2 siRNA, followed by MPP^+^ exposure. The expression of p-Akt1 and p-mTORC1 was determined using Western blotting. The expression of p53, Bax, Bcl-2, and caspase-3 was evaluated using qRT-PCR. Cleaved caspase-3 levels and Annexin V/propidium iodide flow cytometry were used to determine apoptosis. The effects of PI3K inhibition were examined. The results showed that eEF1A2 siRNA significantly reduced the eEF1A2 expression induced by MPP^+^. MPP^+^ treatment activated Akt1 and mTORC1; however, eEF1A2 knockdown suppressed this activation. In eEF1A2-knockdown cells, MPP^+^ treatment increased the expression of p53 and caspase-3 mRNA levels as well as increased apoptotic cell death when compared to MPP^+^ treatment alone. In cells exposed to MPP^+^, upstream inhibition of the Akt/mTOR pathway, by either LY294002 or wortmannin, inhibited the phosphorylation of Akt1 and mTORC1. Both PI3K inhibitors increased eEF1A2 expression in cells, whether or not they were also treated with MPP^+^. In conclusion, eEF1A2 may function as a neuroprotective factor against MPP^+^, in part by regulating the Akt/mTOR pathway upstream.

## 1. Introduction

The eukaryotic translation elongation factor-1, alpha-2 (eEF1A2) gene encodes the eEF1A2 protein, which plays an important role in protein synthesis by carrying aminoacyl tRNA to the A-site of the ribosome during the elongation process of protein translation and being mediated by the eEF1B2 protein [1]. The other variant form is eEF1A1. This is encoded by the *EEF1A1* gene and shares 92% identity and 98% similarity with the eEF1A2 [2]. eEF1A1 is ubiquitously expressed, whereas eEF1A2 is specifically expressed in the brain, heart, and skeletal muscles [2–4]. Overexpression of eEF1A2 has been observed in prostate cancer and intrahepatic cholangiocarcinoma, including tumors in the ovary, breast, and lung [5–9].

A loss of the eEF1A2 expression due to a 15 kb deletion leads to the development of neurological features such as tremors, loss of muscle bulk, and gait disturbance in mice at three weeks of age (before weaning) [4]. This wasted (*wst*) mutation causes the degeneration of motor neurons in the spinal cord of the homozygous *wst/wst* mice, as shown by the occurrence of neuronal vacuolation and reactive gliosis [10, 11]. A *de novo* recurrent heterozygous mutation in the *EEF1A2* gene has been described in humans, and this leads to early-onset epileptic encephalopathy with severe intellectual disability in affected infants [12]. A homozygous mutation in the eEF1A2 gene has also been reported, causing global developmental delay, failure to thrive, and dilated cardiomyopathy and epilepsy, ultimately leading to death in early childhood [13]. These pieces of evidence suggest that eEF1A2 deficiency affects the neurodevelopmental process.

The molecular mechanism by which the loss or overexpression of eEF1A2 affects the survival of various cell types requires further investigation. Our recent study showed that eEF1A2 knockdown aggravated *α*-synuclein accumulation and reduced mitophagy, which resulted in decreased viability, increased apoptotic nuclei, and increased caspase-3/7 in an SH-SY5Y cell model of Parkinson's disease (PD) [14]. A study of hepatocellular carcinoma (HCC) cells from patients showed that siRNA-mediated knockdown of eEF1A2 expression diminished the activation of both Akt and mTOR signaling, suggesting that eEF1A2 is an upstream inducer of PI3K [15]. The silencing of eEF1A2 using siRNA also reduced cell proliferation and increased apoptosis in human HCC cell lines, mediated through the suppression of the PI3K/Akt/NF-*κ*B pathway [16]. Similar siRNA results have been observed in lung adenocarcinoma cells [17]. The activation of Akt by eEF1A2 was previously supported by an overexpression study in pancreatic and breast cancer cells [18, 19].

Evidence of the role of eEF1A2 in the PI3K/Akt/mTOR pathway in neurons is negligible. Our previous study in a human SH-SY5Y neuroblastoma cell line showed that differentiated SH-SY5Y cells, which had been induced to a neuronal phenotype with retinoic acid, tended to express higher levels of eEF1A2 protein than undifferentiated cells [20]. After exposure to the neurotoxin 1-methyl-4-phenylpyridinium (MPP^+^) to establish a cellular model of PD, the mRNA and protein expression of eEF1A2 increased in both undifferentiated and differentiated SH-SY5Y cells, with relatively greater in the differentiated cells. We observed that the increased *EEF1A2* in SH-SY5Y cells was consistent with increased mRNA and protein levels of Akt and mTOR, including their phosphorylated forms. These results implied increased activation of the PI3K/Akt/mTOR pathway [20].

In this study, we investigated whether the protective role of eEF1A2 in differentiated SH-SY5Y cells treated with MPP^+^ was mediated through the PI3K/Akt/mTOR signaling pathway by using siRNA-mediated eEF1A2 silencing. The results show that eEF1A2 exerts its neuroprotective effects through the Akt/mTOR pathway and certain apoptosis-related genes linked to the pathogenesis of PD.

## 2. Materials and Methods

### 2.1. Chemicals and Reagents

Minimum essential medium (MEM), Ham's F12 nutrient mixture (F12), and fetal bovine serum (FBS) were purchased from Gibco (Gaithersburg, MD, USA). All-*trans* retinoic acid (RA), dimethyl sulfoxide (DMSO), 1-methyl-4-phenylpyridinium (MPP^+^), and 3-(4,5-dimethylthiazol-2-yl)-2,5-diphenyltetrazolium bromide (MTT) were purchased from Sigma-Aldrich (St. Louis, MO, USA). Real-time RT-PCR primers were prepared by Biolegio (Nijmegen, Netherlands). LY294002 and wortmannin were purchased from Cell Signaling (Danvers, MA, USA). All other chemicals used in this study were purchased from Merck Millipore (Danvers, MA, USA) unless otherwise stated.

### 2.2. Cell Culture and Treatments

Human SH-SY5Y neuroblastoma cell line was purchased from American Type Culture Collection (ATCC, CRL-2266) (Manassas, VA, USA). Cells were cultured according to our previously published protocol [21]. Briefly, cells were maintained in a 1 : 1 combination of MEM and F12, containing 10% FBS, 0.1 mM nonessential amino acids, 1.5 g/L sodium bicarbonate, 1 mM sodium pyruvate, 100 U/mL penicillin, and 100 *µ*g/ml streptomycin at 37°C in a 95% humidified incubator with 5% CO_2_. Neuronal differentiation of SH-SY5Y cells was performed according to our published procedure [20]. Briefly, a final concentration of 10 M of RA was added to MEM-F12 24 h after cell plating, and cells were kept in the incubator for 72 h. Following differentiation, cells were either transfected with eEF1A2 siRNA oligonucleotides or exposed to MPP^+^ for further experiments. In line with our previous study [22], 1000 *µ*M MPP^+^ was used for the treatment of differentiated cells in this study. For PI3K inhibitor treatment, LY294002 and wortmannin were solubilized in DMSO to the concentrations of 50 and 1 *µ*M, respectively, and were added to the cells 1 h before MPP^+^ treatment.

### 2.3. Measurement of Neurite Length

SH-SY5Y cells were plated in a 6-well plate. After differentiation in 10 *μ*M RA, morphological changes in cells were observed by phase-contrast microscopy. The length of neurite outgrowth was measured using ImageJ software (National Institute of Health, Bethesda, Maryland, MD). The neurite outgrowths were randomly chosen from 60 neuritic processes in each group for measuring the length.

### 2.4. siRNA Transfection

Small-interference RNA targeted to eEF1A2 (sense: 5ʹ-AGGAGAAGACCCACAUCAATT-3ʹ; antisense: 5ʹ-UUGAUGUGGGUCUUCUCCUTG-3ʹ) and negative-control siRNA were purchased from Qiagen (FlexiTube #1027418 and #1022076, respectively, Qiagen, ML, USA). Transfection reagents included Lipofectamine RNAiMAX (#13778030, Invitrogen, Eugene, OR, USA) along with Opti-MEM (#31985088, Gibco, Gaithersburg, MD, USA). 20 *µ*M siRNA was diluted in 3 *µ*l of Opti-MEM. Optimal transfection conditions were obtained by mixing diluted siRNA with diluted Opti-MEM at a 1 : 1 ratio, and the mixture was incubated for 5 min at room temperature. Differentiated SH-SY5Y cells grown in 6-well plates at 60–70% confluence were transfected with siRNA at the final concentration of 50 pmol to silence eEF1A2. After incubation for 48 h at 37°C, the cells were employed in subsequent experiments. Western blotting was used to assess the efficiency of transfection.

### 2.5. Western Blot Analysis

SH-SY5Y cells were lysed in cold RIPA buffer (50 mM Tris pH 7.4, 150 mM NaCl, 1% Triton X-100, 0.1% SDS, 5 mM EDTA, 1% sodium deoxycholate, 30 mM Na_2_HPO_4_, and 50 mM NaF), followed by centrifugation at 12,000 g for 20 min. The supernatants were collected and kept at −80°C until used. The protein concentrations were determined using a BCA Protein Assay kit (Thermo Fisher Scientific, MA, USA). Samples with equal amounts of protein were loaded onto 6–12% SDS-PAGE, transferred onto a PVDF membrane, and blocked with 5% nonfat dry milk in 1× TBST (Tris-buffered saline containing 0.1% Tween-20) for 2 h. The membranes were incubated with primary antibodies, followed by horseradish peroxidase (HRP)-conjugated secondary antibodies. Immunoreactive proteins were detected using the ECL chemiluminescence system (Thermo Fisher Scientific). Primary antibodies used in this study were mouse anti-eEF1A2 (Abcam, Cambridge, UK), rabbit anti-pan-Akt (Cell Signaling, MA, USA), rabbit anti-phospho-Akt1 (Ser473) (Cell Signaling), rabbit anti-mTOR (Cell Signaling), rabbit anti-phospho-mTORC1 (Ser2448) (Cell Signaling), and rabbit anti-caspase-3 (Cell Signaling). Antibody against mouse *β*-actin (Sigma-Aldrich) was used as a loading control. Secondary antibodies included HRP-labeled anti-rabbit (Abcam, Cambridge, UK) and HRP-labeled anti-mouse IgG (Invitrogen, OR, USA). The band intensity was measured using ImageJ software (National Institutes of Health, Bethesda, Maryland, USA). The relative expression of the targeted proteins to the controls was calculated by normalizing the expression with the corresponding *β*-actin band intensities.

### 2.6. Immunocytochemistry

SH-SY5Y cells were fixed with 4% paraformaldehyde (PFA) at room temperature for 30 min, followed by incubation with permeabilizing solution (0.05% Triton X-100 in PBS) for 30 min. Cells were washed twice with cold PBS and blocked with 0.3% bovine serum albumin (BSA) in PBS with 0.1% Triton X-100 at room temperature for 2 h. Rabbit polyclonal antibody against tyrosine hydroxylase (TH) (Cell Signaling) and mouse polyclonal antibody against eEF1A2 (Abcam) were used and incubated at 4°C overnight. Then, the cells were incubated with fluorescein-conjugated secondary antibody (Abcam) and Alexa 594-conjugated secondary antibody (Cell Signaling) for 2 h. Cells were washed and stained with 1 mg/ml of Hoechst 33258 in PBS for 20 min at room temperature. Coverslips were then mounted with Prolong Diamond Antifade Mountant (ThermoFisher Scientific). Images were taken using a confocal laser-scanning microscope (Olympus FV1000, Olympus, Tokyo, Japan). The fluorescence intensity was quantitated using ImageJ software (National Institute of Health, Bethesda, Maryland, MD) and calculated as the intensity of 40 individual cells, located in the more central area of each image with a scale bar of 25 *µ*m, per experiment after subtraction of the background noise.

### 2.7. Quantitative Real-Time RT-PCR

Total RNA extraction was performed using a PARIS kit (Invitrogen, OR, USA). The RNA concentrations were determined using a NanoDrop spectroanalyzer (Thermo Fisher Scientific Inc., DE, USA). The RNA purity was about 1.8 to 2.1, assessed at 260 nm and 280 nm absorbance. Reverse transcription was performed using the Masterscript kit and RT-PCR System (5 PRIME, MD, USA) using 2 g of total RNA. Amplification of the cDNA was performed using the SYBR FAST qPCR kit (Kapa Biosystems, MA, USA). The *β*-actin gene was used as an internal control. The primers used in this study were as follows [21]: p53: forward 5′-GGAGGTTGTGAGGCGCTGG-3′, reverse 5′-CACGCACCTCAAAGCTGTTC-3′; Bax: forward 5′-TGCAGAGGATGATTGCTGAC-3′, reverse 5′-GAGGACTCCAGCCACAAAGA-3′; Bcl-2: forward 5′-CAGCTGCACCTGACG-3′, reverse 5′-ATGCACCTACCCAGC-3′; caspase-3: forward 5′-ATGGAGAACACTGAAAACTCA-3′, reverse 5′-TTAGTGATAAAAATAGAGTTC 3′; and *β*-actin: forward 5′-CATGTACGTTGCTATCCAGGC-3′, reverse 5′-CTCCTTAATGTCACGCACGAT-3′. The qPCR reaction was performed using an Applied Biosystems 7500 Real-Time PCR system (Applied Biosystems, CA, USA). The setting for the cycling condition was as follows: enzyme activation at 95°C for 3 min, initial denaturation at 95°C for 40 cycles of 3 s, and annealing/extension at 60°C for 1 min [21]. To ensure amplification specificity, melting curve analysis was performed. The threshold cycle (Ct) value was determined using the 7500 Software installed in the Applied Biosystems 7500 Real-Time PCR System. The expression levels of the target genes were calculated by the comparative threshold cycle (2^−ΔΔCt^) method after normalizing to the level of *β*-actin expression.

### 2.8. Cell Viability Assay

Cell viability was determined by an MTT assay. MTT was dissolved in Hank's Balanced Salt Solution (Sigma-Aldrich). SH-SY5Y cells were seeded into a 96-well plate at 1 × 10^4^ cells/well and incubated for 24 h at 37°C in 5% CO_2_. 5 mg/ml of the MTT solution was added to each sample and then incubated for 3 h. The medium was replaced with DMSO. The sample absorbance was determined at 570 and 690 nm using a microplate reader with KC4 Synergy HT Software (BioTek, Shanghai, China).

### 2.9. Nuclear Apoptotic Staining

Cells were fixed with 4% PFA for 30 min at room temperature and washed with PBS. After washing, the cells were stained with 1 *µ*g/mL of Hoechst 33258 (Abcam) for 20 min and mounted in 50% glycerol containing 20 mM citric acid and 50 mM orthophosphate. Nuclear morphology was examined under a laser-scanning confocal microscope (Olympus FV1000, Olympus, Tokyo, Japan).

### 2.10. Annexin V/Propidium Iodide Flow Cytometry

SH-SY5Y cells were stained with Dead Cell Apoptosis kit (Invitrogen, Eugene, OR, USA) and assessed for apoptosis by Annexin V-FITC and PI flow cytometry. Briefly, cells were mixed with 1x Annexin-binding buffer to a concentration of 1 × 10^6^ cells/mL. Each sample was stained with FITC-Annexin V and PI working solution. The cells were incubated at room temperature for 15 min, and 1x Annexin-binding buffer was added. The apoptotic cells were evaluated with the FACSCalibur Flow cytometer (BD Biosciences, CA, USA) by detecting the fluorescence emission at 530 nm and 575 nm. Evaluation of apoptotic cell death included both early (Annexin V-positive and PI-negative) and late (Annexin V-positive and PI-positive) apoptotic cells.

### 2.11. Statistical Analysis

Data, collected from three or more independent experiments, were presented as mean ± SEM. Statistical analysis was performed using GraphPad Prism Software version 5 (GraphPad Software, San Diego, CA). One-way analysis of variance (ANOVA), followed by Turkey's post hoc tests for multiple comparisons, was used to test overall statistical significance. A statistically significant difference was considered as *p* < 0.05.

## 3. Results

### 3.1. Verification of Neuronal Differentiation of SH-SY5Y Cells

**Figure 1:**
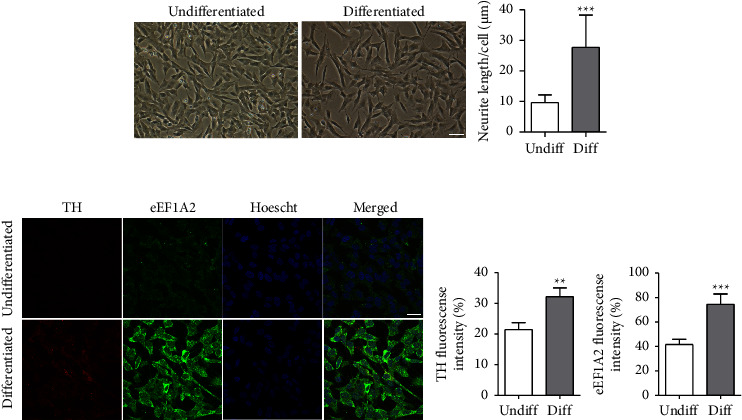
Differentiation of SH-SY5Y cells. (a) SH-SY5Y cells were treated with 10 *μ*M retinoic acid for 3 days. Phase-contrast micrographs shows morphological changes in SH-SY5Y cells after differentiation. Graph represents the length of neurite outgrowths of differentiated cells. Scale bar = 40 *µ*m. Data are expressed as mean ± SD. ^*∗∗∗*^*p* < 0.001. (b) Confocal immunofluorescence micrographs showing the expression of the TH and eEF1A2 proteins in differentiated SH-SY5Y cells. Scale bar = 25 *µ*m. Graphs represent the fluorescence intensity of TH and eEF1A2. Data are expressed as mean ± SEM from three experiments with triplicates each. ^*∗∗*^*p* < 0.01, ^*∗∗∗*^*p* < 0.001. Hoechst, nuclear stain; eEF1A2, eukaryotic elongation factor alpha 2; TH, tyrosine hydroxylase.

The morphology of SH-SY5Y human neuroblastoma cells was determined under a phase-contrast microscope. After 3 days of 10 *μ*M RA treatment, SH-SY5Y cells became differentiated neurons, as shown by an increase in pyramidal-shaped cell bodies and the extension of neurite processes (Figure 1(a)), compared with undifferentiated control cells. The measurement of the average neurite length, indicated by neurite extending more than twice the length of the cell body, showed that differentiated cells had neurite lengths significantly longer than those of undifferentiated cells (27.64 ± 1.4 versus 9.62 ± 0.33 *μ*m; *p* < 0.001). Differentiation of SH-SY5Y cells was also determined by a significantly increased intensity of TH, a marker for dopaminergic neurons, compared to undifferentiated cells, as shown by immunofluorescence (Figure 1(b)). Interestingly, the fluorescence intensity of eEF1A2 was significantly increased in neuronlike cells along with TH when compared to undifferentiated cells.

### 3.2. eEF1A2 Knockdown Reduces eEF1A2 Expression Induced by MPP^+^

**Figure 2:**
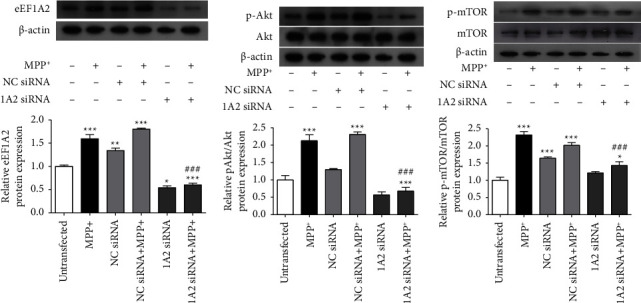
eEF1A2 knockdown reduces eEF1A2 expression and reduces phosphorylation of Akt1 and mTORC1 induced by MPP^+^. Western blot assays were performed in SH-SY5Y neuronal cells after eEF1A2 siRNA transfection and MPP^+^ treatment. (a) Immunoblots of eEF1A2 protein. (b) Immunoblots of phospho-Akt1 protein. (c) Immunoblots of phospho-mTORC1 protein. The density of the bands was normalized with that of *β*-actin protein. Data are expressed as mean ± SEM (*n* = 3). ^*∗*^*p* < 0.05, ^*∗∗*^*p* < 0.01, ^*∗∗∗*^*p* < 0.001 versus untransfected control; ^###^*p* < 0.001 versus MPP^+^. NC, negative control; 1A2, eEF1A2.

To determine the effectiveness of eEF1A2 siRNA transfection on the suppression of eEF1A2 protein in SH-SY5Y cells exposed to MPP^+^, the expression of eEF1A2 protein was examined using Western blot analysis. Based on our previous studies, an MTT experiment indicated that a 50% reduction in cell viability required 1000 *µ*M of MPP^+^ [22]. We used this concentration of MPP^+^ for the treatment of SH-SY5Y cells in this study. In cells with eEF1A2 siRNA alone, the expression of eEF1A2 was significantly decreased compared to the untransfected control (*p* < 0.05; Figure 2(a)), while treatment with MPP^+^ alone for 24 h significantly increased the eEF1A2 protein, compared to the control (*p* < 0.001). However, in cells with eEF1A2 knockdown, the expression of eEF1A2 induced by MPP^+^ was significantly decreased when compared to cells with MPP^+^ treatment alone, as well as the untransfected control (*p* < 0.001).

### 3.3. eEF1A2 Knockdown Reduces Akt and mTOR Phosphorylation Induced by MPP^+^

Our previous study found that MPP^+^ treatment slightly increased the phosphorylation of Akt1 and mTORC1 in SH-SY5Y neuronal cells [20]. To determine how eEF1A2 may play a role in the Akt/mTOR signaling pathway in this Parkinson's disease model, we performed eEF1A2 silencing in SH-SY5Y cells before MPP^+^ treatment. Analysis of protein expression by Western blots revealed that the phosphorylation of Akt1 and mTORC1 was not affected by eEF1A2 knockdown alone when compared to the untransfected control (Figures 2(b) and 2(c)). Exposure of SH-SY5Y neuronal cells with eEF1A2 knockdown to MPP^+^ significantly increased the phosphorylation of Akt1 (Figure 2(b)) and mTORC1 (Figure 2(c)) compared with the eEF1A2 siRNA alone group (*p* < 0.001). It is interesting to note that, in contrast to cells treated with MPP^+^ alone, cells with low eEF1A2 significantly decreased the phosphorylation of Akt1 and mTORC1 induced by MPP^+^ (*p* < 0.001). These findings suggest that low eEF1A2 levels could suppress the activation of Akt1 and mTORC1 in neuronal cells in response to MPP^+^.

### 3.4. eEF1A2 Knockdown Increases p53 and Caspase-3 mRNA Expression Induced by MPP^+^

**Figure 3:**
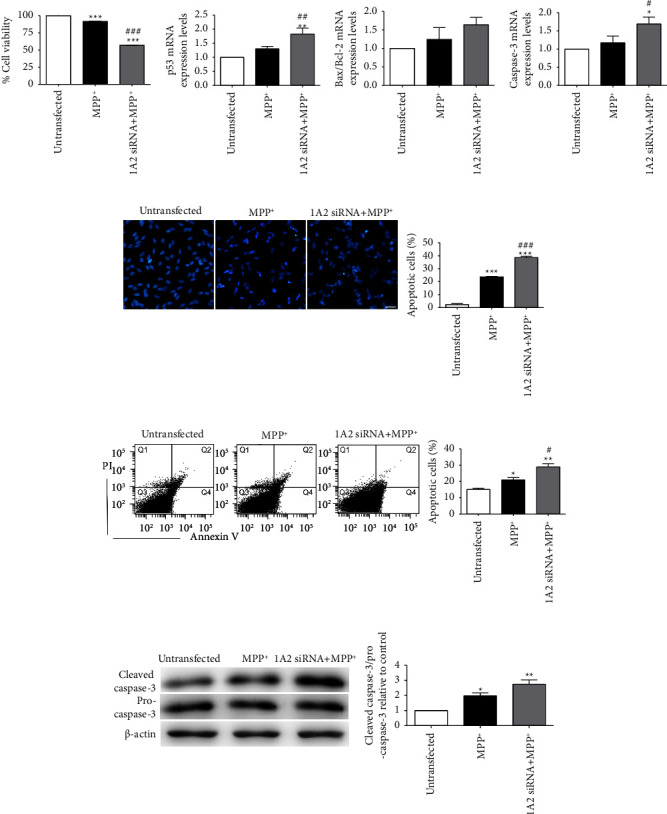
Effects of eEF1A2 knockdown on apoptosis induced by MPP^+^ in SH-SY5Y neuronal cells. (a) Cell viability using an MTT assay. (b–d) Expression of p53, Bax/Bcl-2 ratio, and caspase-3 using quantitative real-time PCR. The expression levels of the mRNAs were normalized to the expression level of *β*-actin. (e) Apoptotic nuclear morphology observed using Hoechst 33258 staining. Scale bar = 30 *µ*m. Graph represents the percentage of cells with apoptotic nuclei. (f) Graphs represent the percentage of Annexin V-positive cells obtained by Annexin V/PI flow cytometry. (g) Graph represents the relative ratio of cleaved caspase-3 to procaspase-3 protein expression obtained from Western blot assay. The density of the bands was normalized with that of *β*-actin protein. Data are represented as mean ± SEM from three experiments with triplicates each. ^*∗*^*p* < 0.05, ^*∗∗*^*p* < 0.01, and ^*∗∗∗*^*p* < 0.001 versus untransfected control; ^#^*p* < 0.05, ^##^*p* < 0.01, and ^###^*p* < 0.001 versus MPP^+^ treatment alone. 1A2, eEF1A2.

Previous studies have found that the inhibition of the Akt/mTOR signaling pathway promotes apoptosis [16, 17]. An MTT assay was performed to determine the viability of SH-SY5Y neuronal cells. However, treatment with MPP^+^ alone significantly decreased the viability of cells to 92%, compared to the untreated control (*p* < 0.001), and cells with eEF1A2 knockdown before MPP^+^ treatment showed a substantial drop in viability to 57%, when compared to MPP^+^ alone (*p* < 0.001) (Figure 3(a)). Next, we investigated three apoptosis-related markers at the mRNA levels using quantitative real-time PCR (Figures 3(b)–3(d)). Although treatment with MPP^+^ alone did not increase the expression of these apoptosis-related markers, the p53 and caspase-3 mRNAs were significantly increased in eEF1A2-knockdown cells treated with MPP^+^ compared to the MPP^+^ alone group (Figures 3(b) and 3(d)). Further assessment of apoptosis showed an increased amount of condensed and fragmented nuclei in the MPP^+^ alone group compared with the control, as shown by Hoechst 33258 staining (Figure 3(e)). eEF1A2 knockdown before MPP^+^ treatment significantly augmented the number of apoptotic nuclei compared with MPP^+^ treatment alone (*p* < 0.001). Annexin V/PI flow cytometry showed similar results. As shown in Figure 3(f), flow cytometry analysis showed the staining patterns of live (Annexin V-FITC-/PI-), early apoptotic (Annexin V-FITC+/PI-), and late apoptotic (Annexin V-FITC+/PI+) cells. Quantitative analysis revealed that the proportion of apoptotic cells significantly increased in the eEF1A2 knockdown with MPP^+^ treatment group compared to the MPP^+^ alone group (*p* < 0.05). Western blot analysis of caspase-3 showed that the cleaved caspase-3 to procaspase-3 ratio significantly increased in both eEF1A2 knockdown with MPP^+^ treatment and the MPP^+^ alone groups, compared to the untransfected control (Figure 3(g)). A slight increase was observed in the eEF1A2 knockdown with MPP^+^ treatment, compared to the MPP^+^ alone group. These findings suggest that cells with a lower level of eEF1A2 might be more vulnerable to the toxic effects of MPP^+^.

### 3.5. MPP^+^-Induced Akt/mTOR Phosphorylation Is Aggravated by PI3K Inhibitors

**Figure 4:**
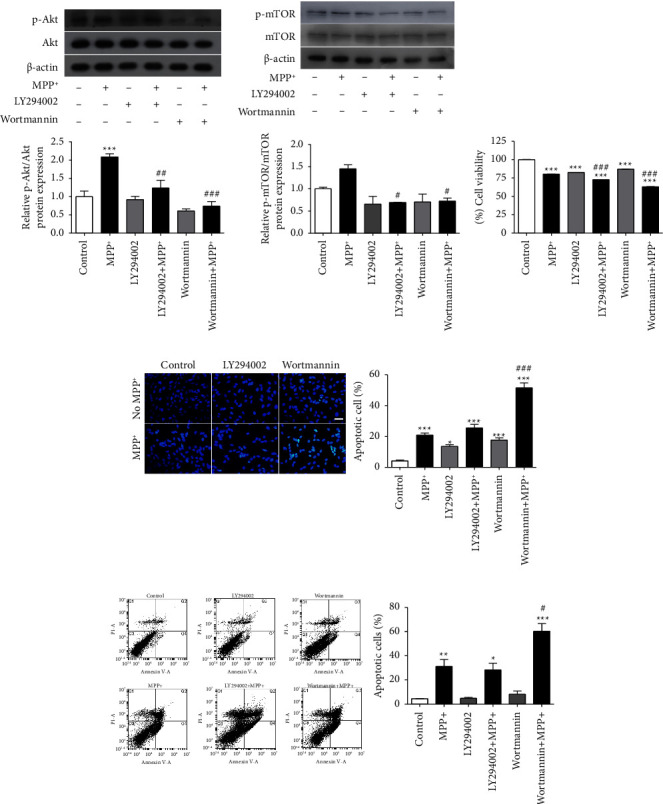
PI3K inhibitors reduce phospho-Akt and phospho-mTOR and potentiate cell death in MPP^+^-treated SH-SY5Y neuronal cells. (a) Immunoblots of phospho-Akt protein. (b) Immunoblots of phospho-mTOR protein. The density of the bands was normalized with that of *β*-actin protein. (c) Cell viability examined using the MTT assay. (d) Apoptotic nuclear morphology observed using Hoechst 33258 staining. Scale bar = 30 *µ*m. Graph represents the percentage of cells with apoptotic nuclei. (e) Graphs represent the percentage of Annexin V-positive cells obtained from Annexin V/PI flow cytometry. Data are expressed as mean ± SEM (*n* = 3). ^*∗*^*p* < 0.05, ^*∗∗*^*p* < 0.01, ^*∗∗∗*^*p* < 0.001 versus control; ^#^*p* < 0.05, ^##^*p* <  0.01, ^###^*p* < 0.001 versus MPP^+^ treatment.

The earlier findings in this study indicated that eEF1A2 knockdown reduced the increment of Akt and mTOR phosphorylation induced by MPP^+^. We then performed further verification on the upstream regulator of the Akt/mTOR pathway using phosphoinositide 3-kinase (PI3K) inhibitors. SH-SY5Y neuronal cells were pretreated with 50 *µ*M of LY294002 or 1 *µ*M of wortmannin for 1 h, followed by exposure to 1000 *µ*M of MPP^+^ for 24 h. The results showed that, in cells exposed to MPP^+^ after pretreatment with LY294002 or wortmannin, the phosphorylation of Akt and mTOR was significantly decreased compared to MPP^+^ treatment alone (Figures 4(a) and 4(b)). As expected, cell viabilities were significantly decreased in the PI3K-inhibitor pretreatment groups, followed by MPP^+^ compared with the MPP^+^ treatment alone (*p* < 0.001; Figure 4(c)). Furthermore, Hoechst nuclear staining showed that the number of apoptotic cells was markedly increased in wortmannin-pretreated cells exposed to MPP^+^ compared to MPP^+^ treatment alone (*p* < 0.001; Figure 4(d)). To confirm apoptotic cell death, Annexin V-FITC and PI flow cytometry were performed. Similar to nuclear staining, the proportion of apoptotic cells significantly increased in wortmannin-pretreated cells exposed to MPP^+^ compared to MPP^+^ treatment alone (*p* < 0.05; Figure 4(e)).

### 3.6. PI3K Inhibitors Increase eEF1A2 Expression

**Figure 5:**
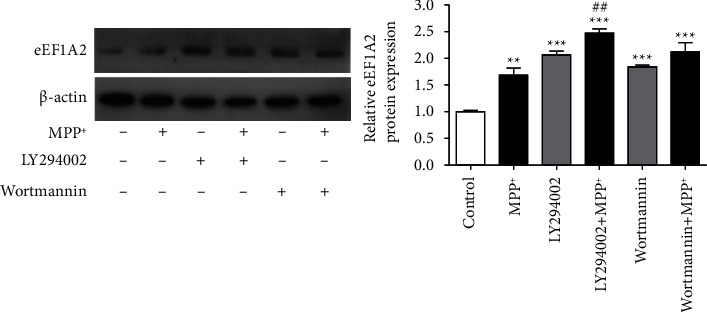
PI3K inhibitors increase eEF1A2 expression. SH-SY5Y neuronal cells were pretreated with 50 *μ*M LY294002 or 1 *μ*M wortmannin for 1 h followed by exposure to 1000 *μ*M of MPP^+^ for 24 h. Western blot assay was used to detect the expression of eEF1A2. The density of the bands was normalized with that of *β*-actin protein. Data are expressed as mean ± SEM (*n* = 3). ^*∗∗∗*^*p* < 0.001 versus control; ^##^*p* < 0.01 versus MPP^+^.

Our earlier results showed that both eEF1A2 knockdown and PI3K inhibitors inhibited the phosphorylation of Akt and mTOR in SH-SY5Y cells when exposed to MPP^+^. However, the interplay between eEF1A2 and the PI3K/Akt/mTOR pathway remains unclear. To investigate whether PI3K signaling affected eEF1A2 expression in neuronal cells, especially those with MPP^+^ exposure, SH-SY5Y neuronal cells were pretreated with 50 *µ*M of LY294002 or 1 of *µ*M wortmannin for 1 h, followed by exposure to 1000 *µ*M of MPP^+^ for 24 h. Western blotting results showed that treatment with LY294002 or wortmannin alone significantly increased eEF1A2 expression compared with the control *p* < 0.001 (Figure 5). In cells pretreated with a PI3K inhibitor followed by MPP^+^, the eEF1A2 expression was also significantly increased compared to the control (*p* < 0.001). Our results indicated that the inhibition of the Akt/mTOR pathway by PI3K inhibitors increased eEF1A2 expression in neuronal cells regardless of MPP^+^ exposure.

## 4. Discussion

Using siRNA-mediated eEF1A2 silencing, our results showed that the eEF1A2 knockdown decreased the viability of neurons and potentiated apoptosis of the cells when exposed to the MPP^+^ neurotoxin, which was mediated in part through the inhibition of the PI3K/Akt/mTOR pathway, as shown by a reduction in the phosphorylation levels of Akt and mTOR. Differentiated SH-SY5Y neuroblastoma cells treated with MPP^+^ were used in the study as a cellular model for PD [23]. There is a debate about whether differentiated or undifferentiated SH-SY5Y cells are better suited. However, our previous study on cell susceptibility to MPP^+^ and the level of expression of apoptosis-related genes and other studies using a 6-hydroxydopamine (6-OHDA) model of PD suggested that differentiated cells may be more suitable for studying the molecular and cellular mechanisms of PD [22, 24]. Our results showed an increase in pyramidal-shaped cell bodies and the extension of neurite processes of retinoic acid-treated SH-SY5Y cells, together with increased fluorescence intensity of TH, suggesting differentiation of the cells into neuronal cells. This neuronal differentiation was associated with an increase in the eEF1A2 protein, as shown in the fluorescent staining, which is similar to our previous observations on the protein blotting [20, 25, 26]. eEF1A2 is highly expressed in postmitotic cells, and studies showed that eEF1A2 might be required for neuronal differentiation of SH-SY5Y cells [26]. Thus, these eEF1A2-positive SH-SY5Y cells were used as target cells for eEF1A2 siRNA transfection in this study. The siRNA sequence that we used effectively silenced the expression of eEF1A2 protein levels.

Treatment with MPP^+^ increased the eEF1A2 protein in differentiated SH-SY5Y neuronal cells, concurring with our previous studies [14, 20]. In this study, we chose 1000 *μ*M of MPP^+^ and a 24 h duration of exposure based on our previous work [22]. Previously, we found that MPP^+^-susceptible cells required 500 and 1000 *μ*M of MPP^+^ for 24 h exposure for undifferentiated and RA-differentiated cells, respectively, to demonstrate a drop in viability to about 50% [20]. Moreover, Martins et al. found that after 24 h of exposure to 500 and 1000 *µ*M MPP^+^, considerable cell death of RA-differentiated SH-SY5Y cells was seen, with no obvious increase in the cell death when the exposure duration was extended to 48 h [27]. The increased eEF1A2 in response to MPP^+^ treatment may reflect a cellular response to increased synthesis of several intracellular proteins involving pro- and antisurvival processes. MPP^+^ itself can induce the activation of the PI3K/Akt/mTOR pathway in SH-SY5Y cells [20, 28]. This study showed that eEF1A2 knockdown diminished this response to MPP^+^ exposure, which could lead to insufficient protective processes and, eventually, neuronal cell death. To support this hypothesis, we investigated the effect of eEF1A2 silencing on the Akt/mTOR signaling pathway. Activation of this pathway is essential for the control of neuronal development, proliferation, maturation, and synaptogenesis [29–31]. According to studies, the PI3K/Akt signaling pathway is in charge of activating the expression of late phase genes involved in neurogenesis, neuronal differentiation, and neuroprotection [32, 33]. The knockdown of eEF1A2 in our study may promote neuronal cell death and increase the vulnerability of neuronal cells to MPP^+^ because of the impairment of PI3K-mediated neuroprotective mechanisms.

The phosphorylation of Akt protein was slightly decreased by eEF1A2 knockdown. Previous studies in human breast cancer cells [19] using eEF1A2 overexpression experiments and siRNA, and HCC cells [15] using eEF1A2 siRNA, have shown that eEF1A2 is a key activator of the prosurvival Akt. In this study, we demonstrated that eEF1A2 might also play a similar role in the regulation of Akt in SH-SY5Y neuronal cells. Previous studies as well as our present work used a phospho-Akt1 (Ser-473) antibody, which can also bind to analogous residues on Akt2 and Akt3 [15, 19]. While Akt2 is mostly expressed in insulin-responsive tissues (such as the heart, skeletal muscle, adipose tissue, and testes), Akt1 is widely distributed and Akt3 is highly expressed in the brain and testes [34]. Research on cortical neurons using specific shRNAs for Akt1, Akt2, and Akt3 showed that Akt2 and Akt3 play distinct and nonredundant roles in cell survival through the regulation of apoptotic pathways [35]. Thus, the inactivation of the phosphorylation of Akt in eEF1A2-silenced neuronal cells, as shown in this study, either with or without MPP^+^ treatment could explain the marked reduction in viability and the increased apoptotic death of these cells.

Investigation on mTOR showed consistent results with those of Akt. The function of mTORC1 is tightly regulated by PI3K/Akt [36]; therefore, we examined the protein expression of mTORC1. The phosphorylation of mTORC1 was not significantly affected by eEF1A2 knockdown alone, but its phosphorylation was markedly decreased in eEF1A2-knockdown cells exposed to MPP^+^ compared with MPP^+^ treatment alone. mTORC1 is associated with the eukaryotic initiation factor 3 (eIF3) complexes, which regulate protein translation initiation [37]. eEF1A is a protein abundantly represented in the eIF3 complex [38]. Previous studies have shown that both mTORC1 inhibition and mTORC2 inhibition enhance apoptosis [39, 40]. Altogether, our findings suggest that eEF1A2 knockdown might act as an upstream regulator of mTORC1 by suppression of its phosphorylation induced by MPP^+^, which subsequently leads to apoptosis.

Our cell viability results showed that eEF1A2 knockdown promoted the death of SH-SY5Y neuronal cells. Such death was augmented in eEF1A2-silenced cells exposed to MPP^+^. The p53 and caspase-3 mRNAs were prominently upregulated in eEF1A2-knockdown cells treated with MPP^+^, as well as a slight increase in cleaved caspase-3. A previous study showed that eEF1A2 siRNA knockdown in human HCC cells inhibited the PI3K/Akt/mTOR pathway and reactivated the p53 protein [15]. Another study in prostate cancer DU-145 and PC-3 cells also showed that eEF1A2 silencing increased the caspase-3 protein accompanied by an enhanced apoptosis rate [41]. Our findings suggest that the suppression of eEF1A2 in SH-SY5Y neuronal cells triggered apoptotic pathways when the cells were exposed to MPP^+^.

Previous studies suggested that eEF1A2 is an upstream inducer of the PI3K/Akt/mTOR pathway [15, 16]. Our results indicated that eEF1A2 knockdown alleviated the increment of Akt/mTOR phosphorylation induced by MPP^+^. Nevertheless, the interplay between the role of eEF1A2 and the PI3K/Akt/mTOR pathway remains unclear. With PI3K inhibitor treatments, we found that both the reversible inhibitor LY294002 and irreversible inhibitor wortmannin inhibited Akt and mTOR phosphorylation induced by MPP^+^, while both inhibitors increased the expression of eEF1A2 protein. Despite increased eEF1A2 levels, PI3K inhibitor-induced cell death was increased. A recent study in MPP^+^-treated SH-SY5Y cells also found that a PI3K inhibitor LY294002 potentiated apoptosis induced by MPP^+^, which was accompanied by increased inhibition of phosphorylation of both PI3K and Akt [42]. Activation of the PI3K/Akt pathway has been shown to inhibit neuronal apoptosis and promote the survival of dopaminergic neurons in a rat model of PD [43]. In contrast, inhibition of the PI3K pathway could lead to neuronal death, which might be mediated through the inhibition of JNK [44]. In response to stress, JNK phosphorylates eEF1A2 [45]. Thus, the inhibition of JNK by PI3K inhibitors may alleviate the function of eEF1A2. Our results suggested that eEF1A2 might be essential for the survival of neuronal cells, especially when the cells were exposed to the neurotoxin MPP^+^. However, the expression level of eEF1A2, which is required for effective neuroprotection, remains to be investigated. This should include studies on eEF1A2 overexpression as well as cellular or animal models that express constitutively active Akt. A recent study has shown that overexpression of eEF1A2 activates the Akt/mTOR pathway, which increases spinal cord neuron survival and reduces inflammation [46]. Research is still needed to determine whether the increased eEF1A2, induced by the PI3K inhibitors, could be a positive feedback mechanism or an adaptive compensatory process for neuroprotection.

## 5. Conclusions

**Figure 6:**
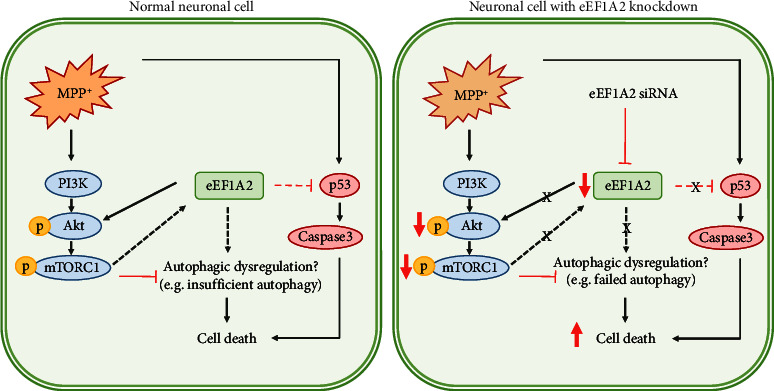
Conceptualized diagram of the effect of eEF1A2 knockdown on the Akt/mTOR pathway in MPP^+^-treated SH-SY5Y neuronal cells.

In conclusion, the results showed that eEF1A2 knockdown can suppress Akt/mTOR phosphorylation induced by MPP^+^, upregulate p53 and caspase-3 expression, and eventually potentiate the apoptotic death of MPP^+^-treated neurons (Figure 6). Similar effects of eEF1A2 knockdown and PI3K inhibitors further support our findings that eEF1A2 may act upstream of the Akt/mTOR pathway. The present study may shed light that modifying eEF1A2 expression could be a possible therapeutic target in PD.

## Data Availability

The data that support the findings of this study are available from the corresponding author upon reasonable request.
